# Why didn’t the TGA consult with Australian researchers and clinicians with experience in psilocybin-assisted psychotherapy for treatment-resistant major depressive disorder?

**DOI:** 10.1177/00048674231172691

**Published:** 2023-05-03

**Authors:** Susan L Rossell, Sally E Meikle, Martin L Williams, David J Castle

**Affiliations:** 1 Centre for Mental Health, School of Health Sciences, Swinburne University of Technology, Hawthorn, VIC, Australia; 2 Department of Psychiatry, St Vincent’s Hospital, Melbourne, VIC, Australia; 3 Melbourne School of Psychological Science, The University of Melbourne, Melbourne, VIC, Australia; 4 Tasmania Centre for Mental Health Service Innovation, University of Tasmania and Statewide Mental Health Service, Hobart, TAS, Australia

On 3 February 2023, the Australian Therapeutic Goods Administration (TGA) announced its Final Decision to down-schedule two psychedelics, MDMA^1^ and psilocybin, specifically for post-traumatic stress disorder (PTSD) and treatment-resistant major depressive disorder (TR-MDD), respectively. From 1 July 2023, the two compounds will be reclassified from Schedule 9 (Prohibited Substances) to Schedule 8 (Controlled Medicines), permitting approved psychiatrists to prescribe them via the Authorised Prescribers pathway. There has been abundant media and general public interest in this decision and experts in the field have been canvassed extensively for their opinion by the media since the announcement.

A puzzling, and concerning, issue is that those experts approached for comment following the decision were certainly not consulted by the TGA, at least in the case of psilocybin-assisted psychotherapy (PAP) for TR-MDD. The authors of the current letter are well qualified to speak to this issue, as they have the only registered active research trial (open-label, *N* = 15) in PAP for MDD, in Australia; a further large (*N* = 160) randomised controlled trial was announced on 1 February 2023 and due to commence mid-year (Meikle et al., 2020; Perkins et al., 2021; Williams et al., 2021).

Why were local experts not consulted? We don’t know.

What would we have said if we had been asked? We would have certainly welcomed the conversation.

While down-scheduling from S9 to S8 may help with the administration of clinical trials, this does not appear to be the motivation behind the TGA decision. Instead, it seems the TGA has yielded to pressure from the public and lobby groups to increase access to these experimental treatments, outside of clinical trials. Despite what the TGA published in its announcement, sufficient levels of evidence have not yet been generated for broad-scale implementation to be justified. Initial results have been promising by most standards, yet many questions remain. These include (but are not limited to): what are the best models of psychotherapeutic care to be delivered alongside the psychedelic dosing; what is the long-term safety profile, especially in terms of psychological recovery and relapse; and how might we differentiate those patients who will benefit from those for whom it could actually be detrimental. Until these questions have been addressed in empirical research, the decision to increase public access outside of clinical trials is questionable, if not concerning.

There are no universally accepted protocols of PAP for TR-MDD. The psychotherapy component is considered absolutely critical to the success of this intervention. The TGA provided no comment on the psychotherapy, likely because there is no consensus on what the therapy should look like. Research is certainly in its infancy in this regard, with many questions remaining as to the psychotherapeutic component.

Who will be an accredited practitioner/authorised prescriber? There was no clarity as to what prerequisites were needed to be a prescriber (e.g. professional background, Australian Health Practitioner Regulation Agency [AHPRA] registration). Psychotherapist training is of utmost importance, but to date, there is a very small cohort of appropriately qualified and experienced therapists and practitioners of PAP in Australia. Setting up recognised (by AHPRA) therapist training programmes will take time and thought and must involve consultation with those with existing experience conducting this therapy in the TR-MDD population. Without training endorsed by regulatory bodies, how will psychotherapy standards, fidelity and safety be monitored? Furthermore, without clear guidelines as to accreditation/authorisation for practitioners, it is uncertain how professional indemnity will be handled by insurance companies.

Many individuals who have undergone PAP have also experienced a significant number of adverse events, and therapists experience many new challenges while conducting this therapy. In our current work to date, adverse events are par for the course, some of them expected, but some of them certainly out of the blue. They require careful consideration and management by a multidisciplinary team of clinicians and researchers. There are no accepted safety manuals or procedures for this at present. How individual clinicians will manage these issues without the added structure, supervision and support of a research trial remains a key concern. Furthermore, given the enormous positive expectations of patients and the public, ensuring that the risks and benefits are accurately and uniformly explained to patients needs careful thought.

In summary, if the TGA had asked us, we would have welcomed the discussion – but we definitely would have suggested more caution than is implied in their current announcement. There are many questions regarding the precise details of this change that remain unanswered. Until these are adequately addressed, we cannot, in good conscience, support this move. We hope the TGA will consider these issues carefully and seek proper consultation in the coming months as these details are worked out.

## Notes

1. Methylenedioxymethamphetamine better known as MDMA is actually an empathogen rather than classic psychedelic. The mislabelling by the TGA perhaps again speaking to poor evidence gathering in relation to this announcement.
